# Investigation of obstructive sleep apnoea syndrome prevalence among long-distance drivers from Zonguldak, Turkey

**DOI:** 10.1186/2049-6958-8-10

**Published:** 2013-02-06

**Authors:** Muhammed E Akkoyunlu, Remzi Altın, Levent Kart, Figen Atalay, Tacettin Örnek, Mehmet Bayram, Meltem Tor

**Affiliations:** 1 Chest Department of Pulmonology, Bezmialem Vakif University, Medical Faculty, Fatih, İstanbul, 34093, Turkey; 2 Department of Pulmonology, Karaelmas University, Medical Faculty, Kozlu, Zonguldak, Turkey

**Keywords:** Obstructive sleep apnoea, Prevalence, Traffic accident

## Abstract

**Background:**

The aim of this study was to assess the prevalence of Obstructive sleep apnoea syndrome (OSAS) in long-distance drivers located in the Zonguldak area and to show the correlation between OSAS and traffic accidents.

**Methods:**

In this study, 241 long-distance drivers who were residents of Zonguldak province were interviewed face-to-face and a questionnaire regarding OSAS symptoms, occupational histories, and numbers of accidents was administered. Body mass measurements were also taken from participants. Patients who exhibited evidence of OSAS underwent polysomnography (PSG).

**Results:**

Snoring was detected in 56% out of all participants, daytime sleepiness was observed in 26.6% and apnoea in 11.6%. All-night PSG was applied to 42 participants who had a high probability of clinical OSAS. Among these, eight had an apnoea-hypopnoea index (AHI) < 5. The prevalence of OSAS was 14.1%. There was a significant relationship between the ratio of traffic accidents per professional years and AHI (r = 0.571; p < 0.005).

**Conclusions:**

OSAS prevalence was higher among long-distance drivers in the Zonguldak region. Disease severity was directly proportional to traffic-accident risk, and thus represents a serious social problem.

## Background

Obstructive sleep apnoea syndrome (OSAS) is a condition of increasing importance because of its neurocognitive and cardiovascular sequelae. As a result of neurocognitive disorders, drivers with OSAS who fall asleep while driving can cause traffic accidents [1]. Turkey has a high number of traffic accidents, nearly half a million per year. Of these, ~4,000 result in fatalities [2].

Reports from around the world provide information regarding the prevalence of OSAS in the general population. However, when comparing the data, differences between methodologies and diagnostic criteria must be taken into account. Some studies used questionnaires to determine sleep apnoea or risk of it, whereas others relied on overnight polysomnography (PSG), the gold standard technique for sleep apnoea diagnosis [3-11]. To our knowledge, no community-based study on OSAS prevalence has been confirmed by PSG in Turkey. We aimed to investigate OSAS prevalence in long-distance drivers in the Zonguldak area and to evaluate the relationship between OSAS and traffic accidents.

## Methods

**Figure 1:**
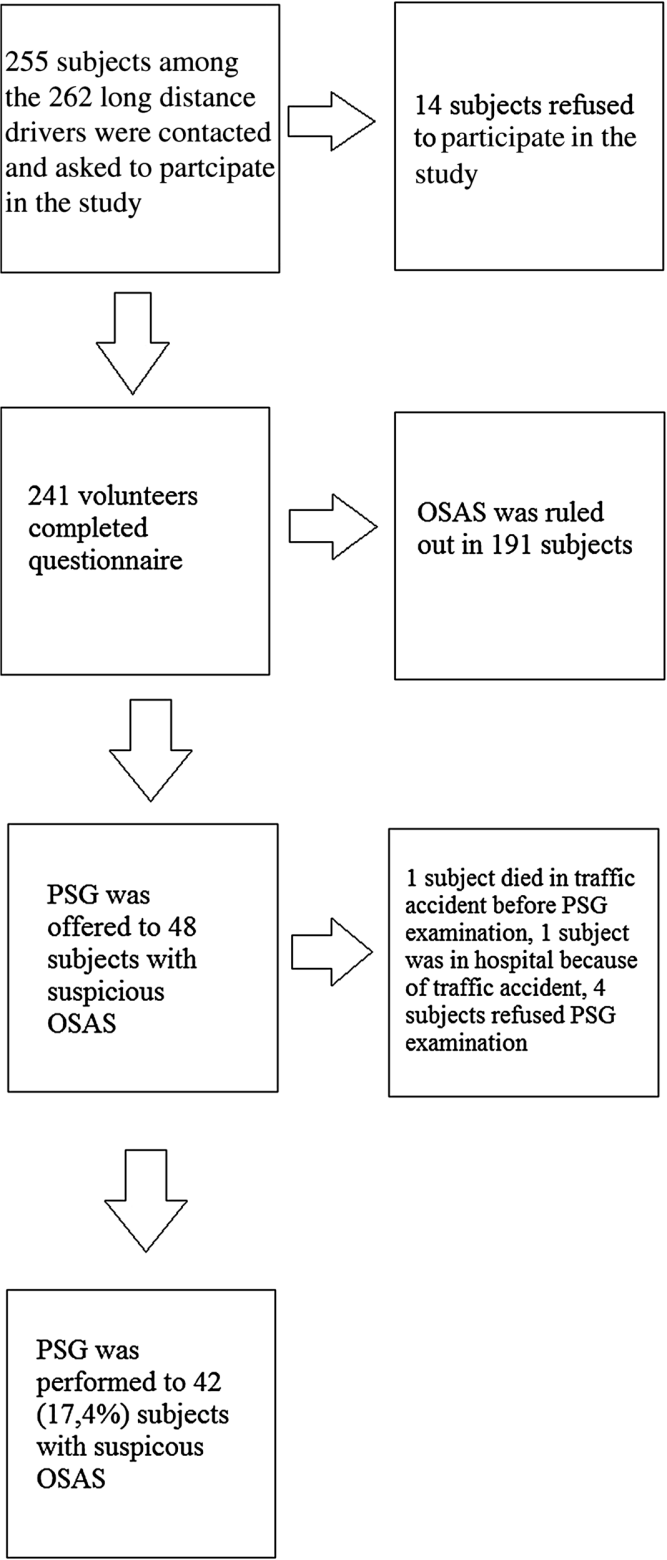
Trial profile.

This cross-sectional study was performed on highway drivers registered at the Zonguldak Chamber of Drivers. The methodology followed in this study is summarized in the flow chart shown in Figure 1.

The study protocol was approved by the Ethics in Research Committee of the University of Zonguldak Karaelmas School of Medicine. After participants provided written informed consent, they were given a questionnaire eliciting information about demographic data, time spent as a long-distance driver, number of traffic accidents, symptoms associated with OSAS, as well as a visual analogue scale (VAS) score for snoring [12], Epworth Sleepiness Scale (ESS) score [13], and a validated Turkish version of the Berlin questionnaire [14]. The weight, height, waist and neck circumference of all participants were measured.

To standardize the rate of accidents among participants with different numbers of employment years, annual accident rates were calculated by dividing the number of accidents by the number of years in the occupation.

Volunteers enrolled in the study were > 18 years of age. Together with habitual snoring, at least one of the following was accepted as the criterion for PSG: excessive daytime sleepiness (EDS), apnoea, at least three positive answers to the nine questions regarding the symptoms of OSAS, ESS score ≥ 8, and neck circumference ≥ 44 cm.

### PSG

Overnight PSG was performed with a computerised system (55 channels; Respironics, USA). Sleep parameters were used according to the standard criteria of Rechtschaffen and Kales [15]. Hypopnoea was defined as a reduction of ≥ 50% in airflow from baseline for at least 90% of the test duration, with ≥1 0 s duration and ≥ 3% desaturation. OSAS was defined as either Apnoea–Hypopnoea Index (AHI) ≥ 5, with associated symptoms such as sleep attacks or EDS, unsatisfying sleep, fatigue or insomnia, witnessed heavy snoring and/or breathing pauses referred by the partner, or as AHI ≥ 15 regardless of associated symptoms [16]. Patients with sleep disorders other than OSAS were excluded, such as upper airway resistance syndrome, periodic leg movement syndrome, or narcolepsy.

### Statistical analysis

Data were analyzed using SPSS, version 11.0 (SPSS, Chicago, IL, USA). Descriptive statistics including frequencies, means ± SD were calculated. Where appropriate, the *χ*^2^ test was used to calculate statistical significance for qualitative variables, while differences between the three groups for quantitative variables were analyzed with the Kruskal – Wallis test with Bonferroni correction for multiple comparisons. In addition, the difference between the presence of OSAS and categorical variables were analyzed by Pearson’s *χ*^2^ test. Differences in OSAS-related parameters between patients with and without OSAS were analyzed by one-way ANOVA. Associations between OSAS cases and PSG measurements were evaluated by the Pearson correlation analysis. Statistical significance was assumed for p < 0.05.

## Results

A total of 262 members of the Zonguldak Chamber of Drivers were contacted, 255 out of which were long-distance drivers. Two hundred and forty-one drivers agreed to participate in the study. The study group was entirely male. Forty-six (19.1%) were bus drivers and 195 (80.9%) were truck drivers. There was no acromegaly or hypothyroidism in the participants, according to a questionnaire-based evaluation and physical examination. Their family histories revealed no members diagnosed with OSAS. Forty-eight (19.92%) participants were never smokers, 143 (59.34%) were current smokers and 50 (20.74%) were former smokers. The mean age, body mass index (BMI), neck circumference, and waist–neck ratio were 42 ± 9.85 years, 27.3 ± 4.2 kg/m^2^, 39.7 ± 2.9 cm, and 0.93 ± 0.4, respectively.

Snoring was detected in 56% of all participants, daytime sleepiness in 26.6%, and apnoea was witnessed in 11.6%. A total of 48 participants with suspicion of OSAS according to symptoms were recommended for PSG, however four of them refused the test, that also could not be performed in other two because one had died in a traffic accident during the study and the other had health problems secondary to a work accident. OSAS was found in 34 (14.1%) out of 42 participants who underwent PSG. Distribution according to AHI score was as follows: 8 (19%) had AHI < 5, 11 (26%) had AHI 5–15, 9 (21%) had AHI 15–30, and 14 (34%) had AHI > 30.

**Figure 2:**
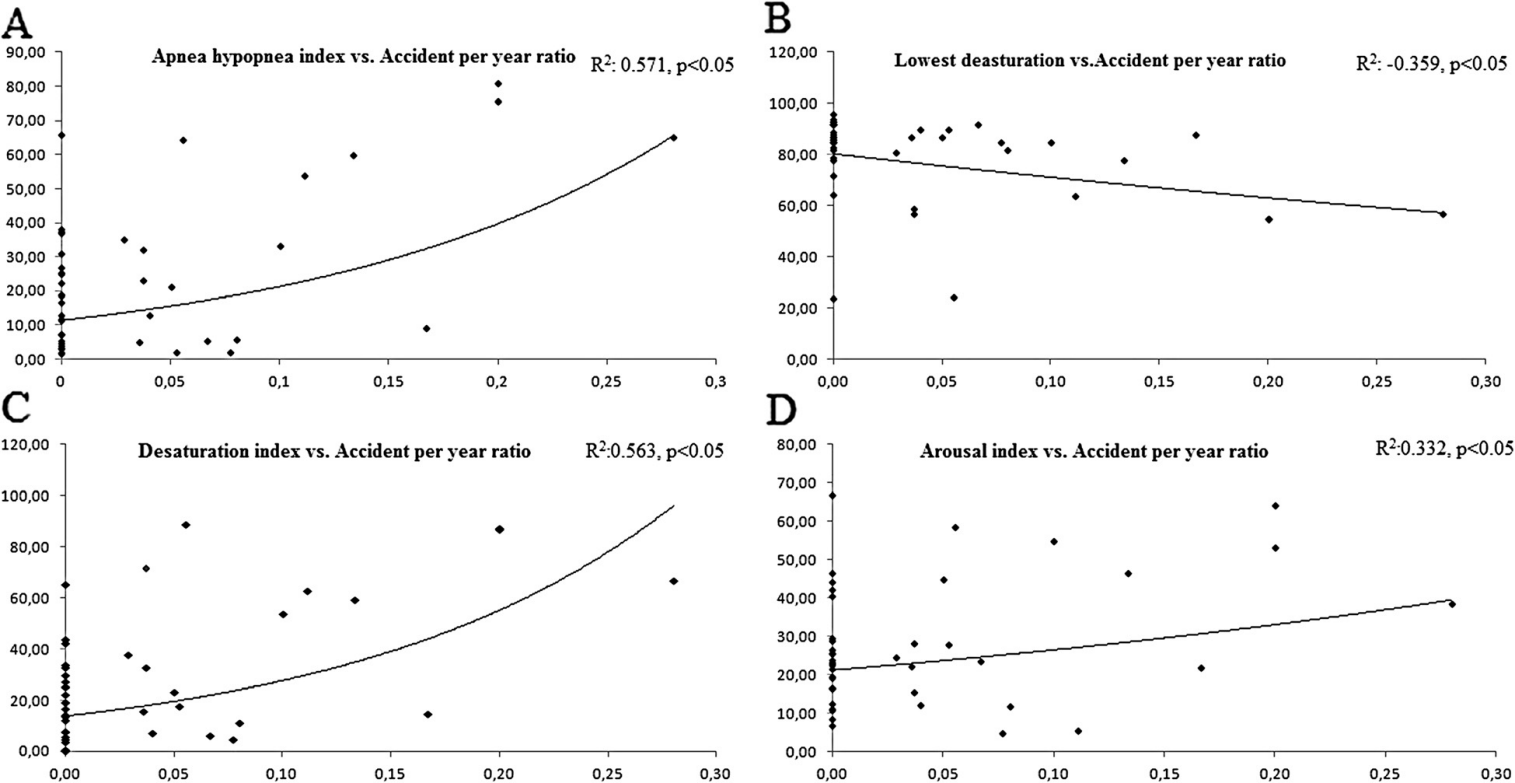
**Graphic of correlations. A** Correlation between lowest saturation with number of accidents per year (r = 0.359, p < 0.05); **B** Correlation between AHI and number of accidents per year (r = 0.571, p < 0.05); **C** Correlation between AHI and number of accidents per year (r = 0.563, p < 0.05); **D** Correlation between arousal and number of accidents per year (r = 0.332, p < 0.05).

Sixty-one (25.6%) of the study participants had a traffic-accident history. The mean accident/year rate was 0.022 in all participants. Age, BMI, neck circumference, VAS for daytime sleepiness, accident/year rate, ESS score, and waist–neck ratios in the OSAS group were significantly higher than subjects with negative symptoms at questionnaire (Table 1). OSAS symptoms are shown in Table 2. We found a significant relationship between the ratio of traffic accidents per year and AHI (r = 0.571, p < 0.05), lowest saturation (r = 0.359, p < 0.05), desaturation index (r = 0.563, p < 0.05), and arousal index (r = 0.332, p < 0.05) (Figure 2).

**Table 1:** Differences in symptoms among study participants

**Symptoms**	**OSAS (+) in PSG**	**OSAS (−) in PSG**	**Negatıve symptoms at questionnaire**	**p**	Intergroup comparison*		
					**p**^**θ**^	**p**^**λ**^	**p**^**ω**^
	**n = 34%**	**n = 8**	**n = 199**				
Witness apnoea	64.7	12.5	1	**<0.001**	**0,015**	**<0.001**	***NS***
Snoring	100	100	45.1	**<0.001**	**NA**	**<0.001**	***0.002***
Awakening during sleep	67.6	50	25.9	**<0.001**	**NS**	**<0.001**	***NS***
Daytime sleepiness	61.8	37.5	18.7	**<0.001**	**NS**	**<0.001**	***NS***
Morning fatigue	73.5	75	48.2	**0.011**	**NS**	**0.009**	***NS***
Character changes	52.9	75	24.9	**<0.001**	**NS**	**0.002**	***NS***
Cognitive disorders	58.8	50	21.2	**<0.001**	**NS**	**<0.001**	***NS***
Mouth dryness	82.4	50	46.1	**<0.001**	**NS**	**<0.001**	***NS***
Sweating	61.8	62.5	38.3	**0.02**	**NS**	**0.014**	***NS***
Nasal obstruction	76.5	75	42.5	**<0.001**	**NS**	**<0.001**	***NS***
Sleepiness while driving	41.2	37.5	14.5	**0.001**	**NS**	**0.001**	***NS***
Sexual dysfunction	41.2	37.5	18.7	**0.009**	**NS**	**0.006**	***NS***

^θ^Represents comparison with OSAS (+) vs. OSAS (−) subjects in PSG; ^ω^ represents comparison with OSAS (+) subjects in PSG vs. subjects with negative symptoms at questionnaire, ^λ^represents comparison with OSAS (−) subjects in PSG vs. subjects with negative symptoms at questionnaire.

**Table 2:** Comparison of OSAS-related parameters in study participants

	**OSAS (+) in PSG**	**OSAS (−) in PSG**	**Negative symptoms at questionnaire**	**p**	**Intergroup comparison**		
					**p**^**ς**^	**p**^**δ**^	**p**^**з**^
	**n = 34**	**N = 8**	**n = 199**				
Age	51 (43–54)	45 (39–50)	40 (33–47)	<0.001	NS	<0.05	NS
Accident/year ratio	0 (0–0.07)	0 (0–0.39)	0 (0–0)	0.003	NS	<0.05	NS
BMI	30 (28–32)	29.2 (28–30)	27 (24–29)	<0.001	NS	<0.05	<0.05
Waist/hip ratio	0.96 (0.91-1.02)	0.91 (0.90-0.92)	0.89 (0.87-0.92)	<0.001	NS	<0.05	NS
Neck circumference	43 (42–45)	41 (39–42)	39 (37–41)	<0.001	NS	<0.05	NS
VAS	7.5 (5–9)	6 (4–8)	1 (0–2)	<0.001	NS	<0.05	<0.05
ESS	7.5 (4–11)	4.5 (3–9)	2 (1–3)	<0.001	NS	<0.05	<0.05

^ς^Represents comparison with OSAS (+) vs. OSAS (−) subjects in PSG; ^δ^ represents comparison with OSAS (+) subjects in PSG vs. subjects with negative symptoms at questionnaire;^з^ represents comparison with OSAS (−) subjects in PSG vs. subjects with negative symptoms at questionnaire. Intergroup comparison was made with Bonferroni correction*.*

## Discussion

The prevalence of OSAS obtained by a combination of questionnaire and PSG among long-distance drivers was 14.1% in our study. This is higher than the estimated 1–4% in the general population in Turkey [17]. This high prevalence in our study could have been due partially to the demographics of our population, which consisted of middle-aged men. It is known that OSAS increases with middle and old age and male sex. OSAS is observed in 2–4% of middle-aged people in the general population [18]. However, the prevalence of OSAS in long-distance drivers in our study was higher than a similar group in the general population [18].

OSAS has been associated with certain features of body shape and abnormality including BMI, waist–neck ratio and neck circumference [9,19]. There was a statistically increase in these alterations in accordance with the literature in our study. The BMI of the non-OSAS group was slightly higher than in the normal population in the same age range in Turkey [20].

Most studies of OSAS prevalence have been based solely on questionnaire data [19]. There have been no studies of the prevalence of OSAS in either the general population or in long-distance drivers using PSG in Turkey [17,20-22]. There has also been a limited number of PSG studies worldwide, and the present study is believed to be the first to examine PSG in long-distance drivers in Turkey [23]. PSG was performed only in participants who were clinically suspected to have OSAS according to the questionnaire. Therefore, asymptomatic OSAS patients might have been classified as non-OSAS, and this might have underestimated the actual prevalence of OSAS.

Our study investigated the major and minor symptoms of OSAS [20,24]. Different results have been reported for the number of major symptoms in national and international studies [17,20,22,24-26]. Snoring and EDS were more common when compared between population-based and driver-specific studies similar to ours. The remaining data of our study group were similar to those of similar populations in the literature.

OSAS symptoms in the OSAS group were significantly higher than in the non-OSAS group. This finding showed that OSAS symptoms queries were effective to predict OSAS.

The prevalence of OSAS based on PSG in long-distance drivers is 8.3-44.3% [25-28]. Nena et al. performed PSG in 22% of 226 train drivers and found OSAS in 13.7% [26]. Prevalence of OSAS, mean age (46.9 ± 3.9 years), mean BMI (28.7 ± 3.7) and methodology were similar to our study. Yusoff et al. detected OSAS in 44.3% of 289 express bus drivers based on PSG in a Malaysian study [28].

OSAS severity is associated with an increased risk of accidents [23,25-28]. The significant correlation between the rate of accidents per year and AHI in the present study supports this relationship. In our study 67.6% of cases had moderate to severe OSAS. A high prevalence of moderate and severe OSAS increases the risk to public health. Our findings suggest that screening of professional drivers for OSAS is important for public health protection. An important social problem associated with OSAS is alteration of cognitive function or occupational and traffic accidents secondary to EDS. In several studies, risk of having traffic accidents was 1.5-6.59 times higher in patients with OSAS [4,8,10,11]. Findley et al. reported that patients with sleep apnoea had a rate of car crash higher than that of all drivers in the state of Colorado, USA [4]. The odds ratio of traffic accidents among the participants with habitual snoring was 1.619 (p = 0.02) in a study based on a questionnaire without PSG [22]. Daytime somnolence and sleep disorders were common in drivers attending the emergency department after accidents in a New Zealand study [11]. Another aspect is that the rate of accidents due to drivers fault may suggest OSAS. Therefore, people who cause an accident should be screened for OSAS.

EDS is not invariably present in patients with OSAS. In a previous study of 23 male patients with EDS and 17 without [29], both groups exhibited a similar AHI. In our study we found a positive correlation between EDS and AHI. Our results may have differed due to the methodology and the definition of EDS. We used only ESS instead of a combination of ESS with multiple sleep latency test (MSLT), as used in the previous study. Drake et al. demonstrated the relationship between accident and sleepiness based on MSLT [30]. They reported that excessively sleepy participants were at significantly greater risk of an accident over a 10-year period compared to their alert counterparts.

The lack of MSLT was one of the limitations of our study. The other limitation was the subjectivity of partner-reported symptoms, and the lack of such for people sleeping alone. However, this is similar in all studies based on questionnaires. Some symptoms, such as somnolence and impotence, were not evaluated with laboratory tests in our study, and this might reduce the diagnostic value of these symptoms and raise the doubt on which questions regarding OSAS should be asked to better select subjects for PSG. Thus, the performance of PSG only in those with suspicion of OSAS according to questionnaire results represents another limitation of our study, because some of the participants with asymptomatic OSAS might have been misclassified as non-OSAS. In light of this, the real prevalence of OSAS among long-distance drivers in Zonguldak may be higher than suggested by our findings. Some traffic accidents are self-reported, which raises the possibility of under-reporting due to fear of adverse consequences. However, our study included a large number of participants, so increasing the confidence in the results.

## Conclusions

In conclusion, OSAS is particularly common in long-distance drivers. We have emphasised the disease severity and the importance of this issue by demonstrating the relationship between OSAS and the rate of traffic accidents. This aspect of OSAS is of more importance to the public than it is the syndrome itself.

## Abbreviations

AHI: Apnoea-hypopnoea index; EDS: Excessive daytime sleepiness; ESS: Epworth Sleepiness Scale; OSAS: Obstructive sleep apnoea; PSG: Polysomnography; NA: Not available; NS: Not significant

## Competing interests

The authors declare that they have no competing interests.
